# Supplementary material for: Survey and Molecular Characterization of Echinococcus granulosus sensu stricto from Livestock and Humans in the Altai Region of Xinjiang, China
Source: Pathogens. 2023 Jan 13;12(1):134. doi: 10.3390/pathogens12010134 (PMC9866323; doi:10.3390/pathogens12010134)
Supplement: Supplementary file 1 [file pathogens-12-00134-s001.zip › Table S1.docx]

| Case number | Race | Sex | Age | Infection organ | Classification of cysts | Location | Date of surgery |
| --- | --- | --- | --- | --- | --- | --- | --- |
| A1074 | Han | Female | 24 | liver | CE3 | Altai | 2018-05-02 |
| A1260 | Mongol | Female | 30 | liver | CE2 | Altai | 2018-06-21 |
| A1296 | Han | Female | 39 | liver | CE4 | Altai | 2017-06-25 |
| A1093 | Han | Male | 20 | liver | CE2 | Altai | 2016-08-06 |
| A1268 | Han | Female | 31 | liver | CE3 | Habahe | 2017-11-14 |
| A1291 | Uygur | Female | 25 | liver | CE4 | Habahe | 2016-02-18 |
| A1286 | Mongol | Male | 46 | liver | CE2 | Habahe | 2018-04-25 |
| A1220 | Han | Male | 49 | liver | CE2 | Habahe | 2018-06-13 |
| A1267 | Uygur | Female | 49 | liver | CE4 | Fuhai | 2018-10-17 |
| A1235 | Uygur | Female | 31 | liver | CE2 | Fuhai | 2017-10-24 |
| A1110 | Han | Male | 48 | liver | CE2 | Fuhai | 2016-01-05 |
| A1889 | Mongol | Female | 39 | liver | CE3 | Fuhai | 2018-01-14 |
| A1226 | Han | Female | 13 | liver | CE2 | Buernjin | 2017-01-20 |
| A1264 | Uygur | Female | 24 | liver | CE2 | Buernjin | 2018-02-14 |
| A1243 | Uygur | Male | 10 | liver | CE3 | Buernjin | 2016-03-07 |

**Supplementary Table S1. Fifteen CE cases information in Altai**
