# Supplementary figures and images for: Survey and Molecular Characterization of Echinococcus granulosus sensu stricto from Livestock and Humans in the Altai Region of Xinjiang, China
Source: Pathogens. 2023 Jan 13;12(1):134. doi: 10.3390/pathogens12010134 (PMC9866323; doi:10.3390/pathogens12010134)

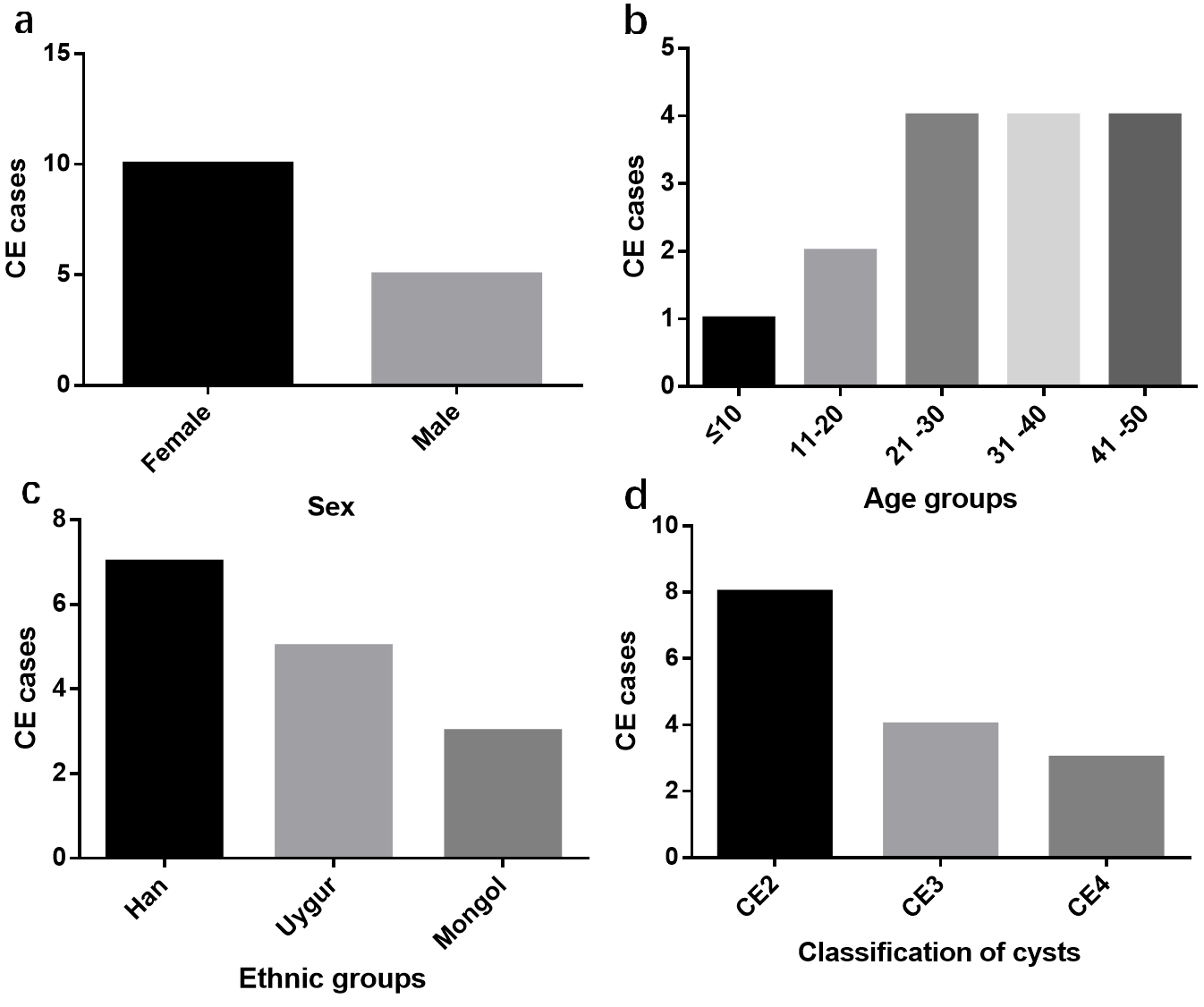

Supplement: Supplementary file 1 [file pathogens-12-00134-s001.zip › Figure S1.tif]
